# Human autoantibodies underlying infectious diseases

**DOI:** 10.1084/jem.20211387

**Published:** 2022-03-23

**Authors:** Anne Puel, Paul Bastard, Jacinta Bustamante, Jean-Laurent Casanova

**Affiliations:** 1 Laboratory of Human Genetics of Infectious Diseases, Necker Branch, Institut national de la santé et de la recherche médicale, Necker Hospital for Sick Children, Paris, France; 2 Imagine Institute, Paris Cité University, Paris, France; 3 St. Giles Laboratory of Human Genetics of Infectious Diseases, Rockefeller Branch, The Rockefeller University, New York, NY; 4 Department of Pediatrics, Necker Hospital for Sick Children, Paris, France; 5 Study Center for Primary Immunodeficiencies, Necker Hospital for Sick Children, Assistance Publique – Hôpitaux de Paris, Paris, France; 6 Howard Hughes Medical Institute, Paris, France

## Abstract

The vast interindividual clinical variability observed in any microbial infection—ranging from silent infection to lethal disease—is increasingly being explained by human genetic and immunological determinants. Autoantibodies neutralizing specific cytokines underlie the same infectious diseases as inborn errors of the corresponding cytokine or response pathway. Autoantibodies against type I IFNs underlie COVID-19 pneumonia and adverse reactions to the live attenuated yellow fever virus vaccine. Autoantibodies against type II IFN underlie severe disease caused by environmental or tuberculous mycobacteria, and other intra-macrophagic microbes. Autoantibodies against IL-17A/F and IL-6 are less common and underlie mucocutaneous candidiasis and staphylococcal diseases, respectively. Inborn errors of and autoantibodies against GM-CSF underlie pulmonary alveolar proteinosis; associated infections are less well characterized. In individual patients, autoantibodies against cytokines preexist infection with the pathogen concerned and underlie the infectious disease. Human antibody-driven autoimmunity can interfere with cytokines that are essential for protective immunity to specific infectious agents but that are otherwise redundant, thereby underlying specific infectious diseases.

## Teaser

Human autoantibodies neutralizing specific cytokines can underlie specific infectious diseases in otherwise healthy patients. The autoantibodies mimic inborn errors of the corresponding cytokines or their response pathways. Four autoimmune phenocopies of inborn errors of cytokine immunity have already been reported.

## Introduction

The clinical consequences of any infection vary greatly between individuals, ranging from silent infection to lethal disease (Casanova and Abel, 2020). The study of single-gene inborn errors of immunity (IEI) has led to the discovery of human genetic and immunological determinants of infectious diseases (Casanova and Abel, 2020; Casanova and Abel, 2021a; Casanova and Abel, 2021b). Most of the more than 450 IEI genetically characterized since 1985 confer a predisposition to infectious diseases (Bousfiha et al., 2020; Tangye et al., 2020; Tangye et al., 2021). Following their discovery in the 1950s, each IEI was thought to underlie various infectious diseases in individual patients. However, from 1996 onward, some IEI were found to underlie a single, specific infectious disease (Notarangelo et al., 2020). These IEI include inborn errors of specific cytokines or their response pathways disrupting immunity to specific microorganisms (Casanova and Abel, 2021a; Casanova and Abel, 2021b; Notarangelo et al., 2020; Tangye et al., 2020; Tangye et al., 2021). Indeed, otherwise healthy patients vulnerable to weakly virulent mycobacteria (Mendelian susceptibility to mycobacterial disease [MSMD]) and/or to the more virulent *Mycobaterium tuberculosis*, carry inborn errors of IFN-γ, type II IFN immunity (Bustamante, 2020; Yang et al., 2020). Patients with inborn errors of type I IFN immunity each suffer from one or a few viral diseases, including herpes simplex virus 1 encephalitis (Bastard et al., 2020a; Zhang, 2020b), influenza A virus pneumonia (Casanova and Abel, 2021b; Lim et al., 2019; Zhang, 2020a), severe rhinovirus pulmonary diseases (Asgari et al., 2017; Lamborn et al., 2017; Lamborn and Su, 2020), hypoxemic COVID-19 pneumonia (Asano et al., 2021a; Casanova and Abel, 2021b; Zhang et al., 2022; Zhang et al., 2020), or adverse reactions to live-attenuated measles (Hambleton et al., 2013; Hernandez et al., 2019) or yellow fever virus vaccines (Bastard et al., 2021b; Hernandez et al., 2019). Patients with chronic mucocutaneous candidiasis (CMC), which is occasionally associated with staphylococcal disease, carry inborn errors of IL-17A/IL-17F (IL-17A/F; Puel, 2020; Puel et al., 2011). Others, with cutaneous staphylococcal diseases, suffer from inborn errors of IL-6 (Chen et al., 2021a; Puel and Casanova, 2019). Finally, rare inborn errors of GM-CSF, which underlie pulmonary alveolar proteinosis (PAP), have not been associated with infections; yet, nocardiosis and cryptococcosis have been diagnosed in other patients with PAP (Trapnell et al., 2019).

**Figure 1.**
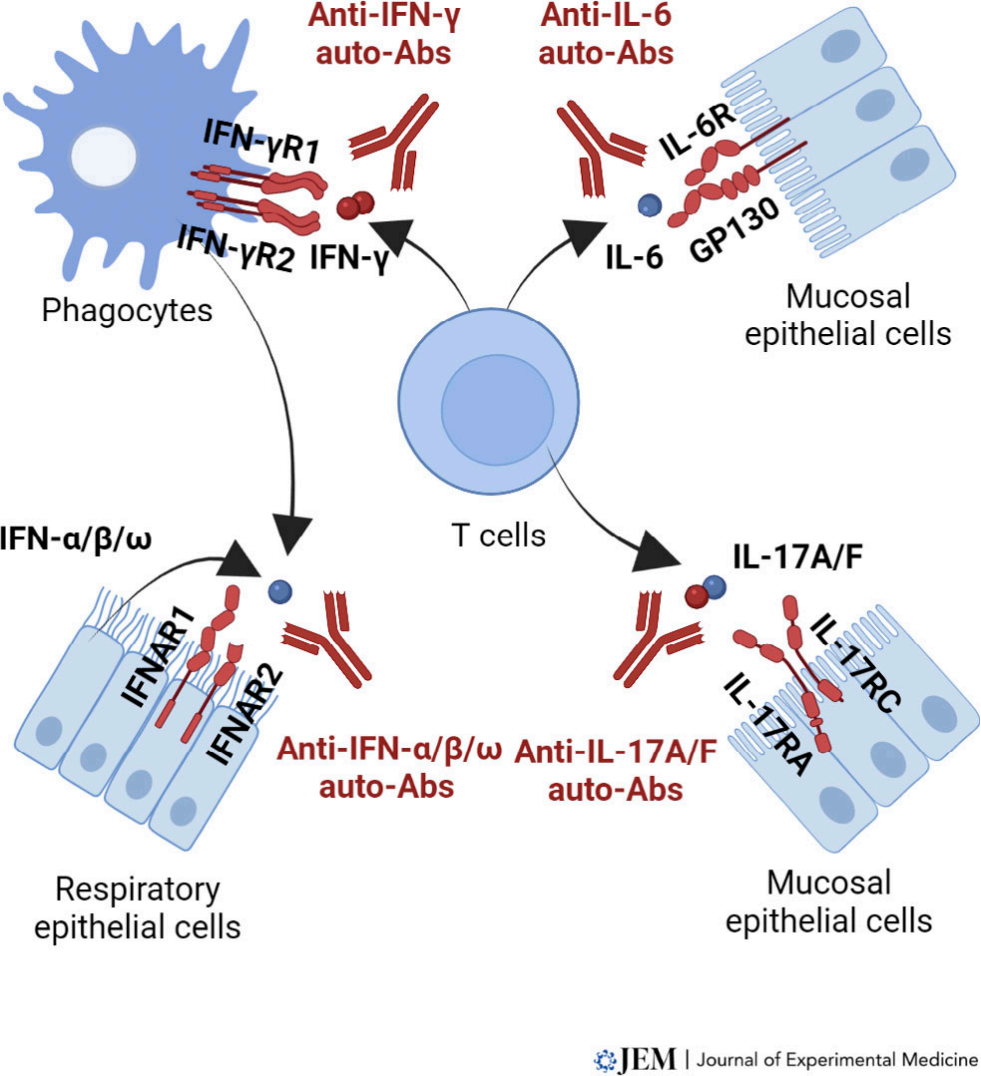
**Human inborn errors of and auto-Abs to cytokines underlying infectious diseases.** This figure illustrates the key actions of four of the cytokines reviewed in this article. Gene products mutated in patients with infectious diseases are shown in red. Auto-Abs neutralizing the cytokines are also shown. For the sake of simplicity, only the most important cell types involved in the biology of each of these cytokines are shown. Molecules, including cytokines and their receptors, are also shown only on key cell types. A more detailed description of the biology of each cytokine can be found in specific reviews.

These IEI preceded (type II IFN, GM-CSF) or followed (type I IFNs, IL-17A/F, IL-6) the discovery of autoantibodies (auto-Abs) neutralizing the corresponding cytokines in patients with the same or a similar infectious phenotype (Ku et al., 2020). By blocking their target cytokines, these auto-Abs underlie infectious phenocopies of inborn errors of the corresponding cytokine or response pathway (Browne, 2014; Ku et al., 2020; Shih et al., 2021; Table 1). Four autoimmune phenocopies of IEI of cytokines have been reported to date (Ku et al., 2020; Tangye et al., 2020; Fig. 1). Auto-Abs against cytokines may underlie mycobacterial disease (type II IFN), one or a few viral diseases (type I IFNs), mucocutaneous candidiasis (IL-17A/F), or staphylococcal disease (IL-6). The infectious diseases of patients with PAP (with no documented inborn error of GM-CSF) include invasive nocardiosis and cryptococcosis, which have also been seen in patients with auto-Abs to GM-CSF (with or without PAP). The pathogenesis of these auto-Abs is largely unknown, but they can be detected in children or adults with IEI underlying broader autoimmunity, such as germline loss-of-function biallelic (or monoallelic) mutations of *AIRE* underlying autoimmune polyendocrine syndrome type 1 (APS-1). Most patients with these auto-Abs have no diagnosed IEI (Bloomfield et al., 2019; Puel et al., 2008). Auto-Abs against cytokines are widely thought to underlie late-onset immunodeficiency (Ku et al., 2020; Tangye et al., 2020), as they are more commonly found in adults, typically with no known underlying IEI (Browne, 2014; Browne et al., 2012a; Browne and Holland, 2010; Ku et al., 2020). Little is known about the causes of these auto-Abs, their prevalence in patients with a given infection and in the general population, the changes in their levels during the life of the individual, their biochemical nature and diversity, their corresponding T and B cell epitopes, and their deleterious or beneficial clinical consequences. We review here the emerging field of anti-cytokine auto-Abs underlying infectious diseases.

**Table 1.** Inborn errors of cytokines or their receptors, their corresponding autoimmune phenocopies (anti-cytokine auto-Abs), and monoclonal antibodies used in therapeutics, together with the associated infectious phenotypes

Cytokine	Receptor of cytokine	Inborn error of immunity	Main infectious disease	Phenocopies (auto-Abs)	Infectious disease	Therapeutic with monoclonal Abs	Infectious disease
Type II IFN (IFN-γ)	IFN-γR1 IFN-γR2	*IFNG* *IFNG*-*R1* *IFNGR2*	- Disseminated *M. bovis*–BCG disease - Disseminated environmental mycobacteria disease	Auto-Abs to IFN-γ	- Disseminated environmental mycobacteria disease - Disseminated *tuberculosis* - Salmonellosis	- Emapalumab - Fontolizumab - AMG811	- Disseminated histoplasmosis - Disseminated salmonellosis
Type I IFNs (IFN-α/β)	IFNAR1 IFNAR2	*IFNAR1* *IFNAR2*	- Herpes virus encephalitis - Severe influenza - Yellow fever - Life-threatening COVID-19 pneumonia	Auto-Abs to IFN-α2, other IFN-α, IFN-β, IFN-ω	- Life-threatening COVID-19 pneumonia - Yellow fever vaccine disease	- Sifalimumab/MEDI545 - Rontalizumab/RG-7415 - AGS-009 - S95021/19D11 -Anifrolimab/MEDI-546	- Respiratory tract infections - Herpes zoster
IL-17A IL-17F	IL-17RA IL-17RC	*IL17F* *IL17RA* *IL17RC*	Chronic mucocutaneous candidiasis	Auto-Abs to IL-17A, IL-17F	- Chronic mucocutaneous candidiasis	- Secukinumab/AIN457 - Ixekizumab/LY2439821 - Brodalumab/AMG 827 - Bimekizumab	- Chronic mucocutaneous candidiasis
IL-6	IL-6R GP130/IL6ST	*IL6R* *IL6ST*	Staphylococcal cutaneous infections	Auto-Abs to IL-6	- Staphylococcal cutaneous infections	- Tocilizumab - Sarilumab - Satralizumab - Sirukumab - Siltuximab	- Staphylococcal cellulitis - Pneumonia by *S. aureus*
GM-CSF^a^	CSF2RA CSF2RB	*CSF2RA* *CSF2RB*	- Nocardiosis? - Cryptococcosis?	Auto-Abs to GM-CSF	- Pulmonary and extra-pulmonary cryptococcosis - Pulmonary and extra-pulmonary nocardiosis	- Lenzilumab - Namilumab - Gimsilumab - Otilimab - Mavrilimumab	- Nasopharyngitis without microbe isolation

a As explained in the text, inborn errors of and auto-Abs to GM-CSF underlie PAP. The infectious diseases seen in these patients are relatively diverse and may result from PAP (including its therapy) and/or from impaired GM-CSF–dependent immunity in alveolar macrophages.

## Mycobacterial diseases in patients with anti–IFN-γ auto-Abs

In humans, IFN-γ is predominantly produced by activated natural killer and T cells (Schoenborn and Wilson, 2007; Yang et al., 2020). IFN-γ, which was first identified as a leukocyte antiviral IFN (Marcus and Salb, 1966), differs from the other IFNs in that it was later shown to be the macrophage-activating factor (Nathan et al., 1983). Human inborn errors of IFN-γ immunity underlie MSMD (Boisson-Dupuis and Bustamante, 2021; Bustamante, 2020). MSMD patients have a selective susceptibility to severe diseases caused by bacillus Calmette-Guérin (BCG) vaccines and environmental mycobacteria (EM). They are typically otherwise healthy and normally resistant to other microbes. The clinical severity of MSMD, ranging from localized to disseminated infections, varies considerably between, and even within affected kindreds, and increases with decreasing levels of IFN-γ activity (Bustamante, 2020; Kerner et al., 2020). Moreover, autosomal recessive (AR) complete IFN-γR1 (Jouanguy et al., 1996; Newport et al., 1996), IFN-γR2 (Dorman and Holland, 1998), and IFN-γ (Kerner et al., 2020) deficiencies are the only known etiologies of MSMD that clearly display complete penetrance in early childhood. Neutralizing anti–IFN-γ auto-Abs (nAIGA) cause an adult-onset and ethnicity-biased immunodeficiency characterized by susceptibility to mycobacterial disease, mostly due to EM (Ku et al., 2020; Shih et al., 2021). Since 2004, at least 500 patients with nAIGAs have been reported (Doffinger et al., 2004; Hoflich et al., 2004; Kampmann et al., 2005; Patel et al., 2005; Shih et al., 2021). All but two of these patients were adults (aged 40–70 yr; Liew et al., 2019), with a balanced sex ratio. These antibodies were initially reported only in patients from South Asia, East Asia, or Southeast Asia, but two patients of European descent were recently reported (Browne et al., 2012a; Hong et al., 2020; Ku et al., 2020; Shih et al., 2021). Over 85% of patients with nAIGAs suffered from disseminated mycobacterial diseases. At least 12 EM species have been isolated from these patients. The more virulent *M. tuberculosis* has also been found in 6% of all patients (Browne et al., 2012a; Kampitak et al., 2011; Shih et al., 2021). However, no infection with *Mycobacterium bovis*–BCG has been reported in these patients, in contrast to those with MSMD, suggesting that the development of auto-Abs occurs after neonatal vaccination. EM disease in patients with nAIGAs is an autoimmune phenocopy of adult MSMD.

## Other infectious diseases in patients with anti–IFN-γ auto-Abs

Other intra-macrophagic infections have also been reported in patients with nAIGAs, with or without mycobacterial diseases. About 18% of all patients with nAIGAs described to date have suffered from infections caused by *Salmonella* spp., *Salmonella* *enteritidis* B, *Salmonella enteritidis* D, and *Salmonella typhi* (Browne et al., 2012a; Shih et al., 2021). Such infections have also been observed in MSMD patients, particularly those with impaired IFN-γ production (Boisson-Dupuis and Bustamante, 2021). Various fungal infectious diseases have also been documented. About 6% of all patients with nAIGAs had cryptococcosis (principally caused by *Cryptococcus neorformans*; Chi et al., 2013; Pithukpakorn et al., 2015; Shih et al., 2021; Wongkulab et al., 2013), 3% had histoplasmosis (*Histoplasma capsulatum*; Pithukpakorn et al., 2015; Shih et al., 2021; Wongkulab et al., 2013), and 22% had talaromycosis (*Talaromyces [Penicillium] marneffei*; Kampitak et al., 2011; Shih et al., 2021; Tang et al., 2010; Wongkulab et al., 2013). In a recent study of 58 HIV-negative adults from South China with severe *T. marneffei* disease, almost 95% had nAIGAs, suggesting a critical role of IFN-γ in immunity to this fungus (Guo et al., 2020; Shih et al., 2021). Shingles was associated with intramacrophagic infections in about 22% of all patients with nAIGAs, suggesting a reactivation of varicella zoster virus. These patients each presented at least one episode of cutaneous shingles, but some displayed reactivation of latent varicella zoster virus infection (Browne et al., 2012a; Chi et al., 2013; Chi et al., 2016; Patel et al., 2005; Shih et al., 2021; Wongkulab et al., 2013). These viral infections have occasionally been seen in patients with AR complete IFN-γR1 or IFN-γR2 deficiency, or autosomal dominant (AD) IFN-γR1 deficiency (Boisson-Dupuis and Bustamante, 2021; Roesler et al., 2011). Thus, not only patients with EM disease but also those with severe infections caused by other intramacrophagic pathogens, such as *T. marneffei*, should be tested for nAIGAs.

## Molecular characterization of anti–IFN-γ auto-Abs

AIGA levels are generally high in the blood of patients producing these antibodies. nAIGAs block cellular responses to up to 200 ng/ml IFN-γ, as shown by the analyses of the phosphorylation of STAT1 (with normal IFN-α–induced STAT1 phosphorylation), the expression of HLA-DR (human leukocyte antigen–DR isotype), the secretion of IL-12 and TNF, and the expression of IFN-γ response genes (Browne et al., 2012a; Krisnawati et al., 2019a; Krisnawati et al., 2019b; Nithichanon et al., 2020; Patel et al., 2005; Shima et al., 2014). Most nAIGAs are of the IgG type, usually of the IgG1, IgG3, and IgG4 subclasses; IgM antibodies, when identified, were not neutralizing, and no IgA antibodies have been detected (Browne et al., 2012a; Wipasa et al., 2018). Other auto-Abs, such as anti–GM-CSF auto-Abs, may also be found in rare patients (Browne et al., 2012a; Kim et al., 2014). Peptide scanning with overlapping peptides covering the human IFN-γ protein has led to the identification of a single major epitope targeted by the nAIGAs in the C-terminal part of IFN-γ (amino acids 121–131, P_121–131_). Amino acids 128–131 (KRKR) are crucial for IFN-γ bioactivity and are conserved across several species. In contrast, amino acids 121–127 are less conserved (SPAAKTG in humans and LPESSLR in mice), and plasma from patients with nAIGAs do not bind mouse IFN-γ, suggesting that their epitope involves amino acids 121–127 (Lin et al., 2016). By binding to the P_121–131_ epitope, nAIGAs neutralize IFN-γ–mediated signaling, a prerequisite for their pathogenicity (Lin et al., 2016). Indeed, nonneutralizing AIGAs in healthy individuals do not target P_121–131_ and do not affect IFN-γ bioactivity (Browne et al., 2012a; Lin et al., 2016). Finally, the P_121–135_ epitope has been shown to have a sequence highly similar to that of the noc2 ribosome assembly protein of *Aspergillus* spp. (Lin et al., 2016; Shih et al., 2021; Wipasa et al., 2018). The identification of a single major B cell epitope displaying similarity to a fungal protein in patients with nAIGAs suggests that molecular mimicry may underlie the development of these auto-Abs. The development of an engineered biologically active variant of IFN-γ, in which the epitope (amino acids 121–127) is modified, enabling it to escape neutralization by nAIGAs, is a possible therapeutic approach that could be considered in patients with nAIGAs (Lin et al., 2016). Various IgG mAbs have been used in clinics (Hommes et al., 2006; Locatelli et al., 2020; Reinisch et al., 2006; Welcher et al., 2015). These antibodies include emapalumab, a human IgG1 mAb, which has been used to treat children and adults with primary hemophagocytic lymphohistiocytosis. Unsurprisingly, albeit rarely, intra-macrophagic infections (e.g., disseminated histoplasmosis, disseminated *Salmonella* group D infection, each found in one patient) have been reported among the most serious adverse reactions (Boedigheimer et al., 2017; Locatelli et al., 2020; Table 1).

## Human genetic underpinnings of anti–IFN-γ auto-Abs

Major human genetic risk factors for nAIGAs have been identified, in the form of specific HLA class II DRB1 and DQB1 alleles found in patients from Southeast Asia, mostly Taiwan and Thailand (Chi et al., 2013; Ku et al., 2016; Pithukpakorn et al., 2015). In a study of 44 Taiwanese patients and 102 controls, a strong enrichment in HLA-DRB1*16:02 (odds ratio [OR] = 8.36, P = 4.5 × 10^−9^) and HLA-DQB1*05:02 (OR = 6.54, P = 1.05 × 10^−8^) was observed in patients (frequencies of 43 and 48%, respectively) relative to controls (frequencies of 8 and 12%, respectively; Ku et al., 2016). A weaker association of nAIGAs was also observed with HLA-DRB1*15:02 and DRQB1*05:01. The same study also investigated a sample of 78 Thai patients and 101 controls and found a strong enrichment in HLA-DRB1*15:02 (OR = 5.33, P = 1.02 × 10^−8^) and HLA-DQB1*05:01 (OR = 4.19, P = 9.61 × 10^−6^) in patients (frequencies of 43 and 33%, respectively) relative to controls (frequencies of 12 and 10%, respectively; Ku et al., 2016). The presence of nAIGAs was also significantly associated with HLA-DRB1*16:02 (OR = 3.80, P = 3.8 × 10^−6^) and DRQB1*05:02 (OR = 2.56, P = 10^−4^). In addition, 100% (*n* = 78) of the Thai patients carried at least DRB1*15:02 or DRB1*16:02. In total, there were 17 homozygotes for DRB1*15:02, 6 homozygotes for DRB1*16:02, and 12 compound heterozygotes for DRB1*15:02/16:02, with ORs for nAIGA carriage ranging from 6.7 to 18.2, whereas the OR in heterozygotes was about 3.6. Similar patterns, but with lower OR, were observed with the two HLA-DQB1 alleles (Ku et al., 2016). Indeed, very strong linkage disequilibrium was observed between the associated DRB1 and DQB1 alleles, underlying two haplotypes, with HLA-DRB1*16:02/DQB1*05:02 common in Asian populations (2.6–18.8%), especially in Taiwan and South China, and HLA-DRB1*15:02/DQB1*05:01 common in Southeast Asia, especially in Thailand (Chi et al., 2013; Ku et al., 2016). HLA-DRB1*15:02 and HLA-DRB1*16:02 are common not only in Southeast Asians but also in Pacific Islanders and Amerindians (for HLA-DRB1*16:02). By contrast, they are rare in Europeans and Africans, in whom HLA-DQB1*05:01 and DQB1*05:02 are more widespread. All Asian patients with nAIGAs identified to date carry at least HLA-DRB1*15:02 or HLA-DRB1*16:02. Interestingly, the only non-Asian HLA-typed patient identified carried neither of these alleles, nor either of the linked HLA-DQB1*05:01 and DQB1*05:02 alleles (Ku et al., 2016). The mechanisms driving the occurrence of auto-Abs in individuals carrying these HLA haplotypes remain unclear, and the corresponding T cell epitopes are unknown.

## Auto-Abs against type I IFNs

Type I IFNs are potent antiviral cytokines that operate as a first line of defense against many viruses (Hertzog and Williams, 2013; Isaacs and Lindenmann, 1957; Isaacs et al., 1957; Lazear et al., 2019; Meyts and Casanova, 2021; Munoz-Moreno et al., 2021). There are 17 type I IFNs (13 IFN-α subtypes, IFN-β, -κ, -ε, and -ω), all closely related phylogenetically, and all binding to the same receptor composed of the IFNAR1 and IFNAR2 chains (Lazear et al., 2019; Manry et al., 2011). Inborn errors of type I IFN immunity underlying severe viral diseases, including influenza and COVID-19 pneumonia, have been described since 2003 (Bastard et al., 2020a; Beck and Aksentijevich, 2020; Bousfiha et al., 2020; Duncan et al., 2015; Duncan et al., 2021; Hambleton et al., 2013; Hernandez et al., 2019; Hernandez et al., 2018; Kreins et al., 2015; Meyts and Casanova, 2021; Zhang et al., 2020). Neutralizing auto-Abs against type I IFNs were first detected in the 1980s, in patients treated with IFN-α or IFN-β for various indications (Rudick et al., 1998; Vallbracht et al., 1981) and in patients with systemic lupus erythematosus (SLE), a condition associated with high levels of type I IFNs in the blood (Gupta et al., 2016; Panem et al., 1982). Auto-Abs against type I IFNs are also observed in patients with thymic abnormalities, such as thymoma (Shiono et al., 2003), or myasthenia gravis (Bello-Rivero et al., 2004; Meager et al., 2003). The production of high levels of anti–type I IFN auto-Abs may be genetically driven and may occur in early childhood, as in patients with APS-1 carrying germline biallelic or monoallelic rare deleterious variants of *AIRE* (Levin, 2006; Meager et al., 2006; Meyer et al., 2016; Oftedal et al., 2015). They are also found in patients with biallelic hypomorphic mutations of *RAG1* or *RAG2* and combined immunodeficiency (Walter et al., 2015), in men with hemizygous mutations of *FOXP3* and IPEX (Rosenberg et al., 2018), and in women with heterozygous null mutations of X-linked *NEMO* and incontinentia pigmenti (Bastard et al., 2020b). In 1984, Ion Gresser described a 77-yr-old woman with disseminated zoster and no history of severe viral disease in whom auto-Abs neutralizing type I IFNs were detected (Pozzetto et al., 1984). Nevertheless, over the last 40 yr, these auto-Abs have generally been considered to be clinically silent.

## Neutralizing auto-Abs against type I IFNs underlie severe or critical COVID-19

In 2020, a large international cohort of patients infected with SARS-CoV-2 was tested for the presence of auto-Abs neutralizing 10 ng/ml IFN-α2 and/or -ω. At least 10% of patients with life-threatening COVID-19 pneumonia carried these neutralizing auto-Abs, which were not found in any of the individuals with asymptomatic or paucisymptomatic infection tested (Bastard et al., 2020b). These auto-Abs were mostly found in men (95%), and half the patients carrying them were over the age of 65 yr (Bastard et al., 2020b). These findings were subsequently widely replicated in independent cohorts (Abers et al., 2021; Acosta-Ampudia et al., 2021; Carapito et al., 2022; Chang et al., 2021; Chauvineau-Grenier et al., 2022; Goncalves et al., 2021; Koning et al., 2021; Raadsen et al., 2022; Solanich et al., 2021; Troya et al., 2021; van der Wijst et al., 2021; Vazquez et al., 2021; Wang et al., 2021; Zhang et al., 2022; Ziegler et al., 2021). Consistently, patients with APS-1 and preexisting anti–type I IFN auto-Abs were found to be at very high risk of severe disease upon SARS-CoV-2 infection (Bastard et al., 2020b; Beccuti et al., 2020; Carpino et al., 2021). In a large study of 22 APS-1 patients, most (*n* = 19, 86%) suffered from severe or critical COVID-19 and four patients died; the others had mild or asymptomatic infections, possibly due to prior or early medical interventions (Bastard et al., 2021c). Another group recently described four younger APS-1 patients with neutralizing auto-Abs, who developed only mild or moderate COVID-19 (Meisel et al., 2021). Overall, patients with APS-1, particularly those over the age of 25 yr, are at very high risk of developing severe or critical COVID-19 pneumonia. They should benefit from early vaccination and prompt treatment in cases of infection before vaccination (Bastard et al., 2021c). There are probably also other conditions, monogenic or otherwise, underlying the production of these auto-Abs and conferring a predisposition to life-threatening COVID-19 pneumonia.

## Neutralizing auto-Abs against type I IFNs in ∼20% of patients with severe or critical COVID-19

The use of more sensitive assays to detect auto-Abs neutralizing more physiological concentrations of type I IFNs (100 pg/ml in plasma diluted 1/10) revealed the presence of such Abs in up to 13.6% of patients of all ages with critical COVID-19. The prevalence of these auto-Abs increased with age, and they were found in more than 20% of patients with critical COVID-19 over the age of 80 yr, and accounted for almost 20% of all COVID-19 deaths (Bastard et al., 2021a). In addition, 6.8% of patients with severe, but not critical COVID-19 also carried such auto-Abs. These data strongly suggested that individuals with these auto-Abs were at higher risk of developing life-threatening disease. Indeed, the highest ORs were obtained for the patients having auto-Abs neutralizing both IFN-α2 and IFN-ω at 10 ng/ml or 100 pg/ml (OR = 67, P < 7.8 × 10^−13^ or OD = 54, P < 10^−13^), whereas ORs were lower, although still highly significant, for individuals carrying auto-Abs against IFN-α2 or IFN-ω alone. Auto-Abs against IFN-β were also found in about 1% of patients with critical COVID-19, with an OR of 5 (P = 0.043). Testing was not performed for auto-Abs against IFN-ε or IFN-κ. In the patients tested, the auto-Abs against type I IFNs were present before SARS-CoV-2 infection, as in patients with APS-1 (Bastard et al., 2021c). Their presence resulted in lower levels of IFN-stimulated gene expression in the blood (van der Wijst et al., 2021), and they were also found in the upper respiratory tract (de Prost et al., 2021; Lopez et al., 2021), along with diminished type I IFN activity in the nasal mucosae (Lopez et al., 2021). It is unknown whether these antibodies are present and functional in the lower respiratory tract. Overall, these data demonstrate that the neutralization of only one subtype or group of type I IFNs (the 13 IFN-α, or IFN-ω, or IFN-β) is sufficient to underlie life-threatening COVID-19 pneumonia (Casanova and Abel, 2021b; Zhang et al., 2022).

## Neutralizing auto-Abs against type I IFNs in the general population

Given the greater risk of severe or critical COVID-19 in individuals with neutralizing auto-Abs against type I IFNs, especially in the elderly (Bastard et al., 2020b; van der Wijst et al., 2021), samples collected from a large general population cohort of over 34,000 individuals aged 20–100 yr before the COVID-19 pandemic were tested. Strikingly, the prevalence of auto-Abs neutralizing 10 ng/ml (and 100 pg/ml) IFN-α and/or IFN-ω increased significantly with age, with 0.17% of individuals under the age of 70 yr tested positive for these antibodies (1.1% for 100 pg/ml), and more than 1.4% of those over the age of 70 yr (4.4%). The prevalence of these antibodies even reached 4.2% (7.1%) for individuals between the ages of 80 and 85 yr. However, it decreased slightly after the age of 85 yr, perhaps because the individuals with these auto-Abs died before the COVID-19 pandemic from other illnesses related to the presence of the auto-Abs. No specific HLA class II alleles have been associated with the production of auto-Abs against type I IFNs. The B and T cell epitopes are unknown. These auto-Abs nevertheless contribute to the increase in the risk of critical COVID-19 in the elderly population. This increase in anti–type I IFN auto-Ab production in the elderly resembles that already described for various other auto-Abs since the 1960s (Hooper et al., 1972; Myasoedova et al., 2020; Parks et al., 2014; Potocka-Plazak et al., 1995; Shu et al., 1975). These auto-Abs appear to have remained clinically silent in these individuals until SARS-CoV-2 infection, although a more complete analysis of the medical history of these individuals is required. Indeed, auto-Abs against type I IFNs were recently shown to underlie severe adverse events following vaccination with the live attenuated yellow fever virus vaccine (Bastard et al., 2021b). It is therefore clearly possible that they may also underlie other severe viral or malignant diseases, especially in the elderly. Various anti-IFN mAbs have been used in clinical practice (e.g., sifalimumab, an anti–IFN-α IgG1 mAb, or anifrolumab, an anti-IFNAR1 mAb, used for the treatment of SLE, in particular), and have occasionally been linked to an increase in the incidence of shingles or respiratory tract infections (Furie et al., 2017; Khamashta et al., 2016; Petri et al., 2013; Tummala et al., 2021; Table 1).

## IL-17A/F and mucocutaneous candidiasis

In humans, the essential and redundant roles of IL-17A/F have emerged through the molecular identification and cellular characterization of inborn errors of IL-17 immunity (Puel, 2020; Puel et al., 2012). Following the identification of STAT3 deficiency as the main genetic cause of AD “classical” hyper IgE syndrome (HIES; Holland et al., 2007; Minegishi et al., 2007; Renner et al., 2007), several teams reported low proportions of Th17 cells in HIES patients (de Beaucoudrey et al., 2008; Ma et al., 2008; Milner et al., 2008; Minegishi et al., 2009). Impaired IL-17 production, possibly due to impaired STAT3-dependent cellular responses to IL-6, IL-21, and/or IL-23 (Zhou and Littman, 2009), has been proposed as an explanation for the recurrent bacterial infections and CMC seen in these patients. Similarly, patients with AR IL-12p40 or IL-12Rβ1 deficiency, and a lack of IL-12 and IL-23 production or of response to these cytokines, respectively, also have abnormally small proportions of Th17 cells, possibly due to the absence of IL-23 signaling (de Beaucoudrey et al., 2008; Ma et al., 2016). These patients typically suffer from MSMD, but about 25% also display CMC (Bustamante, 2020). Finally, about two thirds of patients with AR deficiencies of caspase recruitment domain-containing protein 9—an adaptor acting downstream from the C-type lectin receptors that recognize fungal motifs and lead to the production of pro-inflammatory cytokines, including pro-Th17 cytokines (i.e., IL-6, IL-23)—have low proportions of Th17 cells (Corvilain et al., 2018). Most of these patients suffer from extensive/invasive fungal diseases mostly caused by ascomycete fungi, and about 40% have CMC (Corvilain et al., 2018; Li et al., 2017). These findings suggested a role for IL-17A/F in mucocutaneous protection against *Candida albicans* and, possibly, *Staphylococcus aureus* (Puel et al., 2010b). This role was definitively demonstrated by the identification of 13 inborn errors of IL-17 immunity, including AD IL-17F and JNK1 deficiencies and AR IL-17RA, IL-17RC, and ACT1 deficiencies, in particular, all of which impair or abolish IL-17A/F signaling and are associated with CMC (Puel, 2020). Some defects, such as AR IL-17RA, AR ACT1, and AD JNK1 deficiencies, are also associated with staphylococcal skin diseases (Li et al., 2019).

## Neutralizing auto-Abs against IL-17A/F

CMC is one of the three most common clinical manifestations of APS-1 patients, often the earliest to appear (Cheng and Anderson, 2012). In 2010, two independent studies reported that almost all APS-1 patients, of all ages, tested, had high serum titers of IgG auto-Abs against at least one of the Th17 cytokines (IL-17A, IL-17F, and/or IL-22; Liang et al., 2006; Zheng et al., 2007), neutralizing up to 50 ng/ml IL-17A, 10 ng/ml IL-17F, and/or 0.5 ng/ml IL-22 (in plasma diluted 1/10; Kisand et al., 2010; Puel et al., 2010a). None of the healthy controls, healthy heterozygous relatives, or patients with various endocrine or autoimmune disorders tested in parallel had such auto-Abs (Kisand et al., 2010; Puel et al., 2010a), except two patients with thymoma, who were the only two patients with documented CMC out of the 35 patients with thymoma tested (Kisand et al., 2010). Apart from high levels of auto-Abs against IFN-α and IFN-ω, none of the patients had neutralizing auto-Abs against any of the 13 other cytokines tested (including nAIGAs and known antibodies against cytokines involved in Th17 cell differentiation or maintenance, such as IL-23). A few patients had high levels of auto-Abs against at least one of the three cytokines in the apparent absence of CMC (Kisand et al., 2010; Puel et al., 2010a), but the prevalence and titers of neutralizing auto-Abs were higher in patients with CMC than in those without CMC (Kisand et al., 2010). Auto-Abs against IL-17 cytokines were detectable before the onset of CMC in the informative serum samples of four patients with APS-1 and one with thymoma, with no clear increase in titer after CMC onset, suggesting that they were not triggered by candidiasis (Kisand et al., 2010). No specific HLA class II alleles or haplotypes have been associated with the production of anti–IL-17A/F auto-Abs. The T and B cell epitopes are unknown. Auto-Abs against IL-17A, IL-17F, and/or IL-22 are detected in >90% of patients with APS-1, in whom CMC is a hallmark of the disease (Kisand et al., 2021; Philippot et al., 2021; Puel and Casanova, 2021; Wolff et al., 2013). The lack of staphylococcal skin disease in most APS-I patients may result from residual IL-17A/F immunity, or the compensatory effect of other IL-17 cytokines. The identification of auto-Abs against IL-17A, IL-17F, and/or IL-22 in APS-1 solved the long-standing enigma of CMC in this disorder. The description of mild or moderate oral candidiasis in up to 21% of patients treated with therapeutic Abs blocking IL-17A/F signaling (Reich et al., 2021) was predicted by studies of APS-1 patients (Kisand et al., 2021; Philippot et al., 2021; Puel and Casanova, 2021; Table 1).

## IL-6 and staphylococcal disease

The role of human IL-6, like that of IL-17A/F, has been progressively clarified by the study of IEI impairing its signaling (Chen et al., 2021a; Puel and Casanova, 2019). The description of dominant-negative mutations of *STAT3* as the main cause of AD HIES (Asano et al., 2021b; Minegishi et al., 2007) revealed that impaired STAT3-dependent signaling downstream from several cytokines, including IL-6 (Kane et al., 2014), caused the complex clinical and cellular phenotype observed in these patients characterized by severe early-onset atopic dermatitis, recurrent skin and sino-pulmonary bacterial infections, CMC, poor or delayed clinical and biological signs of inflammation, eosinophilia, high serum IgE levels, low levels of memory B and Th17 cells, and various nonhematopoietic features (Tsilifis et al., 2021). Following this discovery, additional IEI associated with most, if not all of the clinical features observed in classical HIES, were reported, including AR deficiency of ZNF341, a transcription factor governing STAT3 expression and activity (Beziat et al., 2018; Frey-Jakobs et al., 2018), and partial AR (Chen et al., 2021b; Schwerd et al., 2017; Shahin et al., 2019) or AD (Beziat et al., 2020) deficiencies of GP130, the signaling receptor subunit common to all IL-6 family cytokines (Rose-John, 2018), suggesting that impaired IL-6 immunity underlies many of the key immunological and clinical features of HIES. Patients with AR IL-6R deficiency were first reported in 2019 (Nahum et al., 2020; Spencer et al., 2019). These patients displayed most of the clinical features of HIES, including severe atopic dermatitis, recurrent bacterial sinopulmonary infections, recurrent staphylococcal skin abscesses, poor inflammatory responses with undetectable C-reactive protein, high IgE levels, with or without eosinophilia, low-to-normal levels of memory B cells, and low but detectable levels of Th17 cells. These findings suggest that impaired IL-6 signaling drives most of the clinical presentations of HIES, and that this cytokine plays a crucial role in protection against bacterial mucocutaneous diseases, particularly those caused by staphylococci (Puel and Casanova, 2019).

## Neutralizing auto-Abs against IL-6

Four patients with high levels of IgG (IgG1 or IgG4) auto-Abs neutralizing up to 50 ng/ml IL-6 (plasma diluted 1/10) have been reported since 2008 (Bloomfield et al., 2019; Nanki et al., 2013; Puel et al., 2008). These patients (aged from 11 mo to 67 yr) suffered from recurrent staphylococcal subcutaneous abscesses (*n* = 2), *Escherichia coli* and *Streptococcus intermedius* thoracic empyema (*n* = 1), or severe septic shock probably due to *S. aureus* (*n* = 1). None of the patients displayed any detectable increase in serum C-reactive protein concentration during infectious episodes. In the only patient tested, memory B cell counts were low, and serum IgE levels were high (Bloomfield et al., 2019). IL-6 was barely detectable in whole blood from the patients after stimulation, despite normal production by monocytes (as assessed by intracellular staining) or peripheral blood mononuclear cells tested in the absence of patients' plasma (Bloomfield et al., 2019; Puel et al., 2008). B cell epitope mapping was performed with 15-mer peptides overlapping by 10 amino acid residues, generated from the human IL-6 protein sequence. A peptide (LTKLQAQNQWLQDMT) was strongly bound by the serum samples of both patients tested (Nanki et al., 2013). However, no specific HLA class II allele or any other genetic variant, whether germline or somatic, has been associated with the occurrence of neutralizing anti–IL-6 auto-Abs. The T-cell epitope remains unknown. Collectively, inborn errors of the IL-6 pathway and their autoimmune phenocopies suggest that IL-6 is crucial for immunity to bacterial diseases, including staphylococcal skin diseases in particular, and for acute-phase inflammatory responses. Consistent with this conclusion, occasional bacterial skin and sinopulmonary infections, such as cellulitis and pneumonia, possibly caused by *S. aureus*, have been reported in patients following treatment with tocilizumab (a humanized monoclonal Ab against the IL-6 receptor), sirukumab, or siltuximab (anti–IL-6 mAbs; Aletaha et al., 2021; Pawar et al., 2019; Rose-John et al., 2017; Table 1).

## GM-CSF and infections

PAP is a severe lung disease characterized by the accumulation of surfactant lipids and proteins in the alveolar space, resulting in progressive respiratory failure and an increase in the risk of infection (Trapnell et al., 2003). Rare patients with severe early-onset PAP due to inborn errors of the GM-CSF pathway have been reported since 1997, with AR deficiency of the βc receptor chain (Dirksen et al., 1997) common to the receptors for IL-3, IL-5, and GM-CSF, or AR deficiency of GM-CSFRα, encoded by the *CSF2RA* gene (Martinez-Moczygemba et al., 2008; Suzuki et al., 2008; Suzuki et al., 2010). These mutations may impair the terminal differentiation of alveolar macrophages, through impairment of the GM-CSF–dependent induction of expression of the transcription factor PU.1 in these cells, as shown in GM-CSF–deficient mice, resulting in a lower capacity to catabolize surfactant (Trapnell et al., 2019). Several cases of superinfections with unusual pathogens were reported from the early 1960s onward, before the identification of any genetic or immunological cause of PAP, some of which were caused by *Nocardia* spp. (Pascual et al., 1989). In a literature review aiming to identify all reported cases of PAP and unusual infections between 1950 and mid-2010, 75 cases were found, with nocardial infections being the most frequent, identified in *n* = 32 (43%) cases, with *Nocardia asteroides* as the causal agent in 19 cases (Punatar et al., 2012). Most patients suffered from pulmonary nocardiosis (*n* = 24), with or without infections at other sites, including the central nervous system (CNS, *n* = 6; Punatar et al., 2012). Other infections were also reported in these patients: mycobacterial (*n* = 28, 37%) in some, mostly due to *M. tuberculosis* (*n* = 21, 75%), with a few cases of *Mycobacterium kansasii* (*n* = 4, 14%) or *Mycobacterium avium* (*n* = 3, 11%) infections, or fungal in others (*n* = 15, 20%), mostly caused by *Aspergillus* spp. (*n* = 5, 33%), *Cryptococcus* spp. (*n* = 6, 40%), or *Histoplasma capsulatum* (*n* = 4, 27%; Punatar et al., 2012). Human GM-CSF is thus required for the efficient removal of surfactant by alveolar macrophages, and thereby and/or by hitherto unknown mechanisms for pulmonary defense against several pathogens, including *Nocardia* and *Cryptococcus* in particular. Yet, the rare patients with inborn errors of the GM-CSF receptor had PAP but no documented infection. There is therefore no causality between GM-CSF deficiency and nocardiosis or cryptococcosis.

## Neutralizing auto-Abs against GM-CSF

High titers of neutralizing auto-Abs against GM-CSF have been reported in patients with idiopathic PAP worldwide since 1999 (Kitamura et al., 1999; Seymour and Presneill, 2002), in about 90% of the more than 400 PAP cases reported (Trapnell et al., 2019). These patients typically developed symptoms in adulthood (Seymour and Presneill, 2002). In addition to typical respiratory infections, these patients also displayed pulmonary and extrapulmonary (e.g., CNS) infections with various pathogens, including *Nocardia* spp., mycobacteria, *Histoplasma* spp., *Cryptococcus* spp., and *Aspergillus* spp. These infections may be secondary to PAP, the use of steroids, and/or impaired GM-CSF signaling directly compromising alveolar macrophage immunity to these pathogens (Punatar et al., 2012). Since 2013, high titers of IgG (mostly IgG1) auto-Abs neutralizing 10 ng/ml GM-CSF (plasma diluted 1/10) have been found in patients with adult-onset isolated idiopathic disseminated disease mostly due to *Cryptococcus* spp., almost exclusively *Cryptococcus gattii* (Applen Clancey et al., 2019; Browne et al., 2012a; Crum-Cianflone et al., 2017; Demir et al., 2018; Huynh et al., 2020; Kuo et al., 2017; Panackal et al., 2017; Perrineau et al., 2020; Rosen et al., 2013; Saijo et al., 2014; Stevenson et al., 2019; Viola et al., 2021), *Nocardia* spp. (Rosen et al., 2015), or more rarely, *Aspergillus* spp. (Arai et al., 2015). Some of these patients eventually developed PAP after their infectious disease (Rosen et al., 2013). The brain and lungs were the most frequently affected organs. Most of the patients were male (72%) and of various ancestries. A recent genome-wide association study of autoimmune PAP in patients and controls of Japanese ancestry found that the HLA class II allele HLA-DRB1*08:03, which is common in Asian populations (e.g., 8.3% in Japanese individuals) but very rare or absent in other populations, including Europeans (e.g., 0.3% in Germans, 0% in Italians), was associated (P = 0.035) with high levels of anti–GM-CSF auto-Abs in patients, suggesting an underlying genetic predisposition for the production of these auto-Abs, at least in individuals of Asian ancestry (Sakaue et al., 2021). In contrast, no HLA allele was associated with these auto-Abs in a study of 47 European patients with PAP (Anderson et al., 2019).

The T and B cell epitopes remain unknown. Thus, studies of inborn errors of GM-CSF and their autoimmune phenocopies suggest that GM-CSF is a crucial cytokine for immunity to *Nocardia* and *Cryptococcus* spp., particularly in the lungs and CNS, and that patients with idiopathic isolated cryptococcosis or nocardiosis may suffer from inborn errors of the GM-CSF pathway. Several anti–GM-CSF (KB003/lenzilumab, namilumab/AMG203, TJ003234, gimsilumab, otilimab/GSK3196165) or anti–GM-CSFRα (mavrilimumab) mAbs have been developed for clinical use in patients with various conditions (e.g., severe asthma, psoriasis, rheumatoid arthritis, COVID-19, chronic myelomonocytic leukemia). Rare infectious events have been reported (e.g., nasopharyngitis) with a slightly higher incidence than that for the placebo group. However, to our knowledge, no cases with features of PAP have been identified (Molfino et al., 2016; Patnaik et al., 2020; Table 1). Overall, while it is clear that inborn errors of and auto-Abs to GM-CSF underlie PAP, the infections seen in these patients are relatively diverse and may be a consequence of PAP itself (and its consequences, including steroid therapy) and/or of a dysfunction of GM-CSF–dependent immunity, especially in alveolar macrophages.

## Concluding remarks

These studies provide a compelling evidence that autoimmunity may not only be triggered by infection (Bigley and Cooper, 2021; Knight et al., 2021), but that it can predate infection and be causal for infectious disease. The findings reviewed here have direct clinical implications for the diagnosis and management of patients with auto-Abs against any of these four or five cytokines. The depletion of auto-Abs or of the corresponding B cells may prevent relapses of infection, as shown for mycobacterial disease (Browne et al., 2012b; Czaja et al., 2014; Koizumi et al., 2017; Pruetpongpun et al., 2016). The detection of these auto-Abs before the occurrence of disease may lead to specific measures being taken, such as vaccination against the pathogen, or treatment with mAb against the pathogen, or early treatment with exogenous recombinant cytokines (e.g., IFN-β) following infection, as exemplified by COVID-19 (Vinh et al., 2021). The detection of these auto-Abs may also constitute a contraindication for some vaccinations, such as vaccination with live attenuated viruses in patients with auto-Abs against type I IFNs (Bastard et al., 2021b). These studies also raise the possibility that other infectious diseases may be caused by the same or other auto-Abs directed against cytokines. For example, other viral illnesses may develop due to the presence of auto-Abs against type I IFNs, and mycobacterial diseases or diseases caused by intramacrophagic microorganisms may develop due to the presence of auto-Abs against type II IFN (Bastard et al., 2021a; Shih et al., 2021). It will, therefore, be of interest to test for known auto-Abs in various cohorts of patients with idiopathic infectious diseases. Conversely, it may also be useful to test the hypothesis that these auto-Abs protect against some cytokine-dependent conditions (Uggenti et al., 2019). For example, it has been suggested that auto-Abs against type I IFNs are associated with a milder course of SLE (Gupta et al., 2016; Morimoto et al., 2011). The discovery of new auto-Abs directed against cytokines is another important challenge. Broad screening for auto-Abs in patients with infectious diseases would benefit from being performed at least 1 yr after infection, as infections can themselves trigger of the production of various auto-Abs. This search would also benefit from the discovery of new inborn errors of cytokines.

These studies also pose more fundamental biological questions. A first set of questions concerns their causes. The production of auto-Abs against type I IFN and IL-17 may result from IEI impairing T cell tolerance (Kisand et al., 2010; Meager et al., 2006; Puel et al., 2010a), but little is known about the genetic basis of other auto-Abs. All the various IEI underlying type I IFN auto-Abs impair T cell tolerance in the thymus or the periphery (Bastard et al., 2021c; Bastard et al., 2020b; Rosenberg et al., 2018; Walter et al., 2015). The sudden increase in the production of auto-Abs against type I IFNs in individuals over the age of 60 yr is another mystery (Bastard et al., 2021a). This increase may be due to genetic or epigenetic causes, which may be germline or somatic. The distribution of these auto-Abs by age, sex, and ancestry is known only for those directed against type I IFNs (age, sex) and type II IFN (ancestry; Bastard et al., 2021a; Bastard et al., 2020b; Browne et al., 2012a; Shih et al., 2021). It will also be important to determine the distribution of auto-Abs against other cytokines in the general population. The East Asian predominance of auto-Abs against type II IFN reflects the higher frequency of predisposing HLA-DRB1 alleles in these populations (Chi et al., 2013; Ku et al., 2016; Pithukpakorn et al., 2015), raising questions about possible HLA associations for other anti-cytokine auto-Abs, as suggested for anti–GM-CSF auto-Abs in PAP patients of Japanese ancestry (Sakaue et al., 2021). Another fundamental question is the nature of the cytokine-specific Igs and their B cell epitopes, which are known only for auto-Abs against type II IFN (Lin et al., 2016) and IL-6 (Nanki et al., 2013). The high prevalence of auto-Abs against type I IFN in the elderly European population, and in almost all patients with APS-1, together with the diversity of the type I IFNs recognized, suggests that there is unlikely to be an HLA association and implies the existence of multiple T cell epitopes, or a promiscuous T cell epitope. Auto-Abs against IL-17A/F are also found in most APS-1 patients. Investigations of the causes, nature, distribution, and consequences of known and newly discovered auto-Abs against cytokines promise to be an exciting area of study.
